# The Parent: Supported or Supplanted

**Published:** 1917-05-05

**Authors:** 


### SOME PROBLEMS OF TO-DAY.*

### III. The Parent: Supported or Supplanted?

The attention of the nation is called to its loss in child life by voices increasingly numerous and insistent. Now the newspapers announce a Baby Week for July. The President of the National Union of Teachers has been speaking to that important body of wastage "wholesale and profligate," and referring to the 6,000,000 children in their schools as survivors heavily handicapped. We begin to hear as an over-tone the question whether parents are really fit to take charge of their children. On the other side we have the fact, proved by over a century of experiment, that organised institutions do not adequately replace the home as a training school for the science of life. Thus, the vast George Müller orphanages at Bristol, housing 1,000 under one roof, were found to turn out young people of invertebrate character. Dr. Barnardo reduced his numbers from thirty in a "small" home to a number scarcely exceeding that of an ordinary working-class family. Boarding-out is increasingly adopted for the workhouse child. "Even a bad" home, Sir William Chance once declared as the result of his long experience, is better training than "even a good" institution. Much force, no doubt, resides in the adjective. The home, as also the Home, may be good in an ascending scale, or indifferent, or bad. Most of us have noticed how often "Mother used to say—— " appears as a source of action among the best young people. The mind sickens at such a story as that of the slum conversation in which a woman was heard to advise another to bring up her children "as bad as you can, then the good people 'll take the expense of them off you, same 's they done me." No one maintains that it does not matter what the home is like, whether "sweet home" or its pitiful caricature. The schoolmaster's oldest gibe at his "enemy, the parent," is stultified by his anxiety lest his pupils should be allowed to slip their collars at the school gate and go Fantee throughout the long holidays.

Undoubtedly, the worst exploitation of child-labour has been that of parents. In the early years of last century workhouse children were sent in droves by Guardians (alas, for the name!) to North-country mines and Lancashire cotton-mills, to a slavery dignified as apprenticeship. When an Act of 1802 had to order that girls and boys should sleep apart, and not more than two in a bed, and to ordain that the children should be taught something of the three R's and religion, we get a glimpse of indescribably revolting conditions. Yet the parents of the day willingly sent their own children, often mere babies going out foodless in the chill of winter mornings, to share these pitiful gains. It was not till 1833 that another Act limited the factory age to nine years, *except in silk-mills*, where infants were allowed to work up to ten hours a day till the age of thirteen, and officially exempted from all education. Eleven more years passed before the silk-mill child's age was limited to eight. In this year, 1844, two alternative half-time schemes were instituted, but under both the child paid for its education by deductions from its wages. The case of the child chimney-sweeps is better known. It was the increase of crime by discharged apprentices, and not any parental protest, that brought improvement, aided, no doubt, by the spread of better thought. If the demands now made on behalf of popular education seem startling and excessive, it is worth while to learn from what a point the pendulum has swung. Is all well as things are, then? Are we now in danger of going too far in the other direction, of imposing book-learning on children whose bread should be earned by manual labour, and so overcrowding clerical avocations with the incompetent? Are we likely at the same time to undermine parental authority by over-interference from outside? At a recent educational meeting in Essex it was laid down that the elementary scholar must now be more looked after outside the school. One, at least, of the recent educational programmes urges that school meals would be valuable not only for nutrition but as fostering corporate life and good manners. Nursery schools for the child under five are a feature officially notified for the new schemes, and the aim is to diffuse them widely enough to be in reach of every home. If school master and mistress, school doctor and nurse, Care Committee worker and health visitor, Sunday school teacher and club leader, scout-master and country holiday secretary are to undertake each department of the child's life, is it not a hollow mockery to call upon parents to train their own families? Is not the State, as regards the working man's child, rapidly approaching the position of the greedy diner who only took the ends of the pudding, but did so by dividing it in the middle?

The danger lies on the surface, yet, paradoxical as it seems, this kind of action may be the only possible way of rousing the parents to real care for their children. Sir George Newman testifies that some 10 per cent. of elementary scholars, =600,000, are ill-nourished. Well, only the other day in North London a munition worker, whose children ran the street in rags, fed on "doorstep slices," told her neighbours that her life-long ambition for a diamond ring would now be realised. Should we shrink from "interference"? At Brighton, one of the first boroughs to adopt the permissive Act giving the Education Committee power to work as Juvenile Employment Bureaux, Care Committee visitors were amazed at the number of cases in which a mother would reply that boy or girl had got some work, but what it was she didn't rightly know, but it wasn't the same place as last week. The "conscience of the working man," so freely exploited on platforms, has too commonly meant satisfaction that the children are safely herded for much of Sunday and occupied in a harmless and even proper manner, with prizes and treats as natural rewards for their attendance. Must we not use deliberate effort to rouse parents to more real interest? And to participation, for it must be remembered that the home can act as a Penelope, unravelling by night much of the day-woven web.

This, then, is the problem: How to make the new and vigorous State effort for the young citizen a help to the parent, and how to bring parent-power to the side of the State effort, which, without it, must largely be stultified.

Meantime, let us not give less than their due to the thousands of excellent parents who do their best with infinite self-forgetfulness, and who are not to be blamed if they do not always and instantly welcome every new scheme for their children which other people living in other circumstances assure them to be excellent. And if there is still any educated woman who has a little leisure, let her spare a few hours—only a few—in each week to do sorely-needed service, and learn invaluable lessons as a visitor for a Care Committee.

---

* Previous articles appeared March 31 and April 7.

